# Simultaneous resection of gastric and gallbladder metastasis from renal cell carcinoma treated by laparoscopic and endoscopic cooperative surgery: a case report

**DOI:** 10.1186/s40792-019-0569-x

**Published:** 2019-02-04

**Authors:** Osamu Kinoshita, Moyu Dohi, Yusuke Horii, Atsushi Ikai, Tomohito Kitamori, Tetsuro Yamashita

**Affiliations:** 1 Department of Surgery, Maizuru Medical Center, Kyoto, Japan; 2 Department of Gastroenterology, Maizuru Medical Center, Kyoto, Japan; 3 Department of Urology, Maizuru Medical Center, Kyoto, Japan

**Keywords:** Renal cell carcinoma, Gastric metastasis, Laparoscopic and endoscopic cooperative surgery (LECS)

## Abstract

**Background:**

Metastases to the stomach or gallbladder from any malignancy is rarely noted, and simultaneous metastases to both organs are atypical. We present a unique case of simultaneous multifocal metastases of the stomach and gallbladder from renal cell carcinoma (RCC).

**Case presentation:**

The case involved a 60-year-old man, with a past history of RCC (clear cell type, G2, T1b N0 M0 Stage I) treated by a right nephrectomy. Three years after the nephrectomy, a routine gastrointestinal endoscopy found an ulcerative lesion in the greater curvature of the gastric body. The gastric tumor was pathologically proven to be a metastasis from RCC. Furthermore, computed tomography incidentally revealed a mass lesion in the fundus of the gallbladder, which was also diagnosed as a potential metastasis from RCC. As endoscopic ultrasonography of the gastric tumor suggested the tumor potentially invaded to the submucosal layer, gastric wedge resection via a laparoscopic and endoscopic cooperative surgery (LECS) technique was applied to the gastric tumor, and laparoscopic cholecystectomy to the gallbladder tumor was simultaneously performed. Histological examination confirmed that the tumors of the stomach and gallbladder were both metastatic RCC. The hospitalization period after surgery was not eventful, and the patient was discharged on postoperative day 7. Thereafter, the patient required examination every 3 months, did not use anticancer agents, and has survived without relapse to 9 months after the surgery.

**Conclusions:**

For patients with locally resectable RCC metastases, complete metastasectomy may bring long-term tumor control. Moreover, LECS for gastric metastasis is a reasonable approach for minimal invasiveness and an oncologically feasible outcome.

## Background

The incidence of renal cell carcinoma (RCC) has increased over the last decade in the USA and is estimated to be approximately 5% of all cancers [1]. The rich vascular proliferation noted in RCC is thought to be the reason behind increased hematogenous spread leading to distant metastasis. One-third of cases are diagnosed with distant metastasis, for which the 5-year relative survival rate is 12% [1]. The usual sites of RCC metastasis are well known such as the lung, bone, liver, adrenal gland, brain, and contralateral kidney; however, the stomach or gallbladder is rarely noted. Furthermore, simultaneous RCC metastasis to the stomach and gallbladder is an unique situation. Here, we present an unusual case of simultaneous metastasis of the stomach and gallbladder from RCC, treated by laparoscopic and endoscopic cooperative surgery (LECS) for gastric metastasis.

## Case presentation

**Fig. 1:**
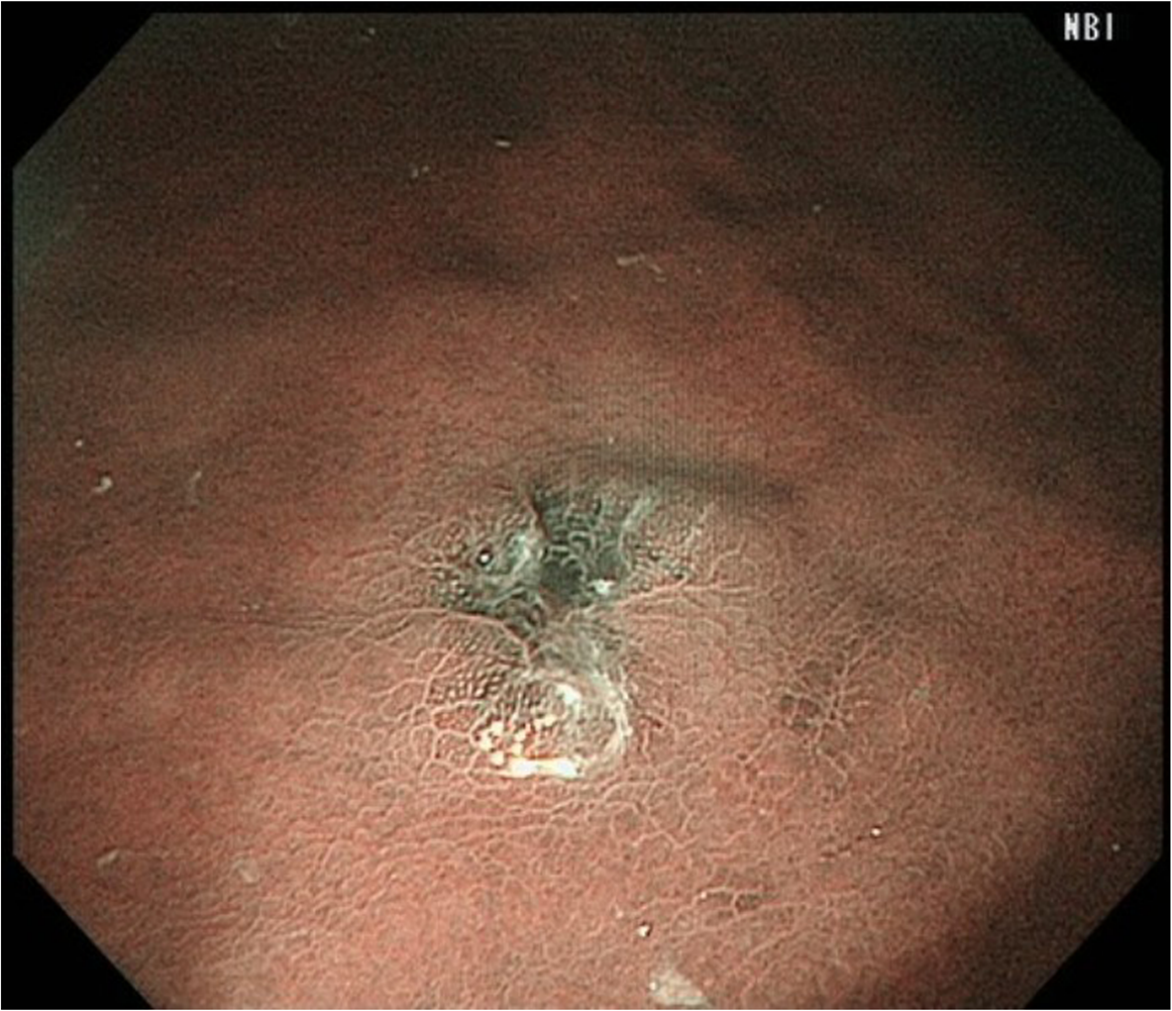
Gastrointestinal endoscopy findings

**Fig. 2:**
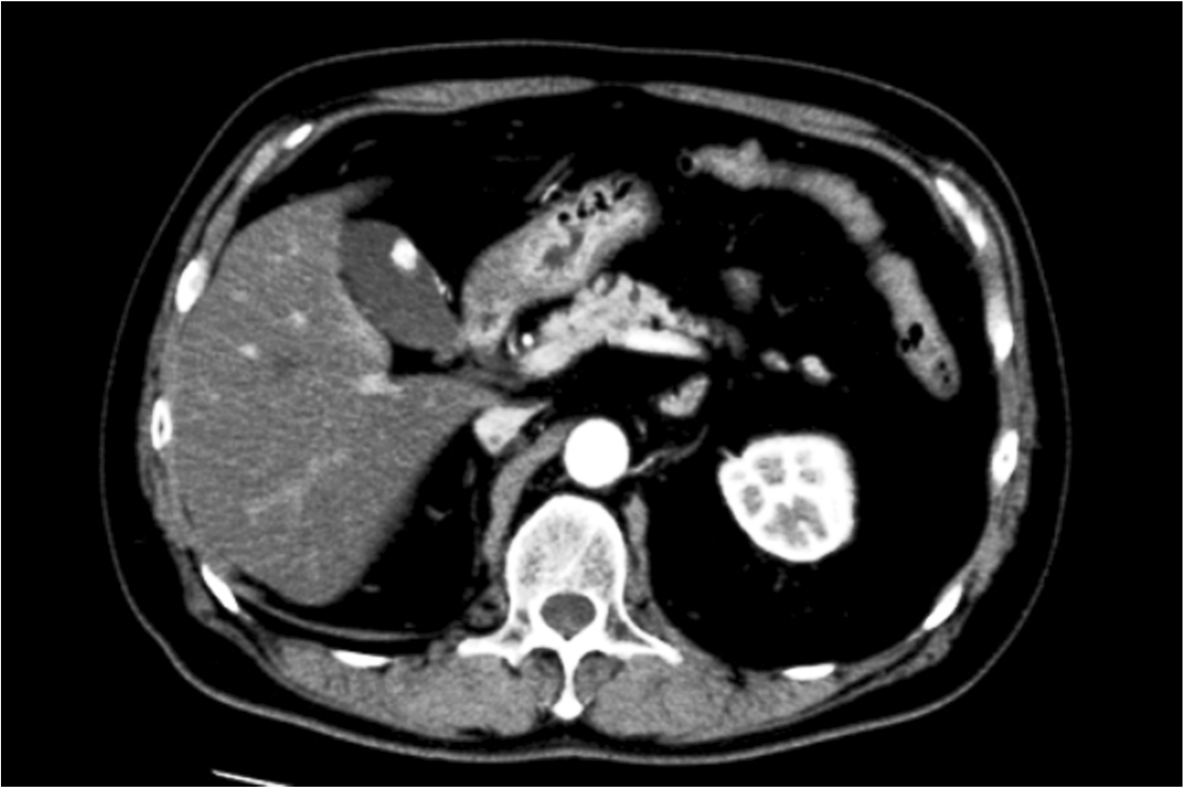
Computed tomography findings

A 60-year-old man with a past history of RCC (clear cell type, G2, T1b N0 M0 Stage I) treated by a right nephrectomy in June 2015 was required to have a follow-up examination at 6-month intervals after surgery, without the use of an anticancer agent. In January 2018, a routine gastrointestinal endoscopy found an ulcerative lesion of approximately 10 mm diameter in the greater curvature of the gastric body (Fig. 1). An endoscopic ultrasonography (EUS) of this lesion showed the first three sonographic layers were blurred, which suggested submucosal invasion. An endoscopic biopsy of the lesion exhibited clear cytoplasm with prominent nucleoli, which was histologically compatible with metastasis to the stomach of the patient’s known RCC. On the other hand, computed tomography (CT) incidentally detected a well contrast-enhancing round-shaped mass in the fundus of the gallbladder (Fig. 2). Additional ultrasonography revealed a sessile polypoid lesion, and gallbladder stone and wall thickening were not observed. Although these findings were lacking conclusive evidence of diagnosis whether the gallbladder tumor was primary or metastatic, the circumstantial evidence potentially pointed to the tumor as a metastasis from the patient’s known RCC. 18F-Fluoro-deoxyglucose positron emission tomography combined with CT (FDG-PET/CT) was performed as a preoperative workup to detect other possible remote metastasis. However, specific FDG uptake was not shown, even in the gastric and gallbladder tumors. The blood examination was unremarkable.

**Fig. 3:**
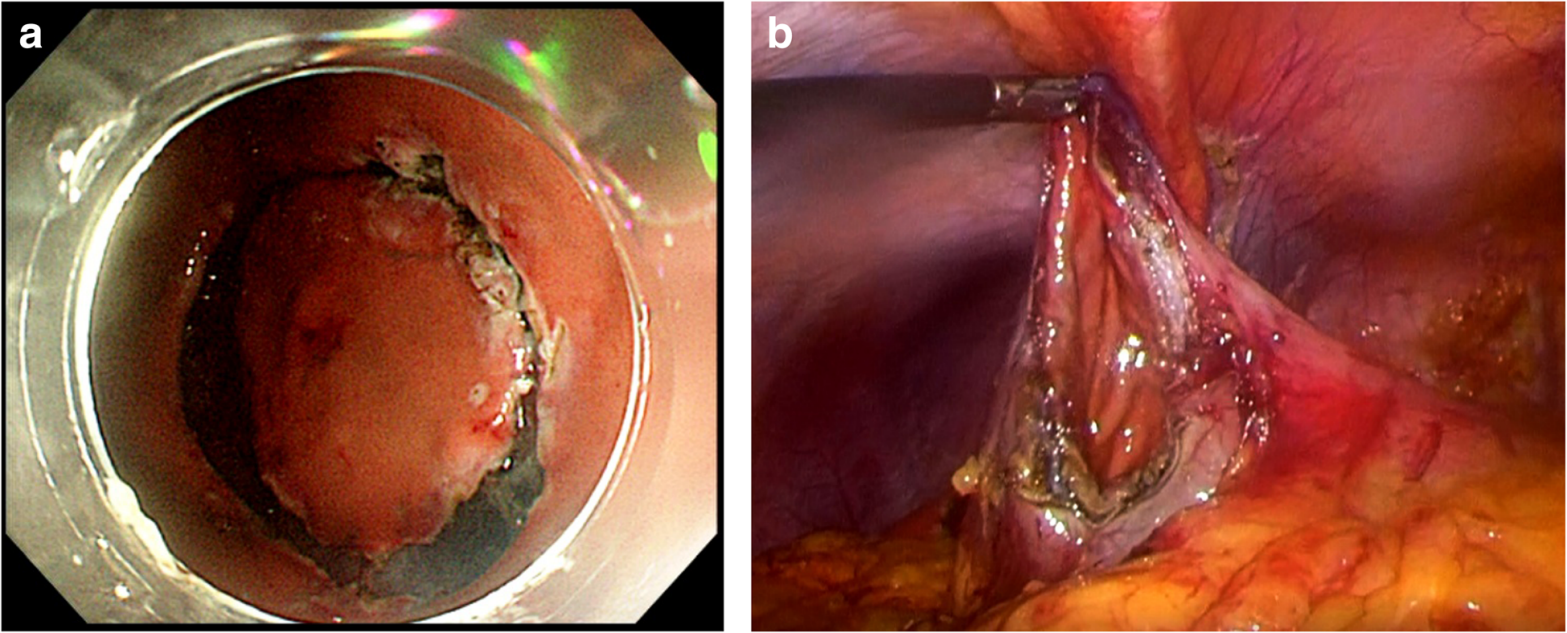
A 2-cm incision was made at the umbilicus, and a wound protector and 12-mm port were inserted for the laparoscopic camera. Thereafter, a 12- and a 5-mm port were inserted at the left and right upper quadrants of the abdomen, respectively, for the working instruments. **a** A submucosal incision around the gastric tumor was made intraluminally through the gastrointestinal endoscope. **b** A seromuscular dissection was laparoscopically made under gastrointestinal endoscopic observation. The specimen was extracted through the umbilical port and the gastric incision hole was sutured using a barbed suture

**Fig. 4:**
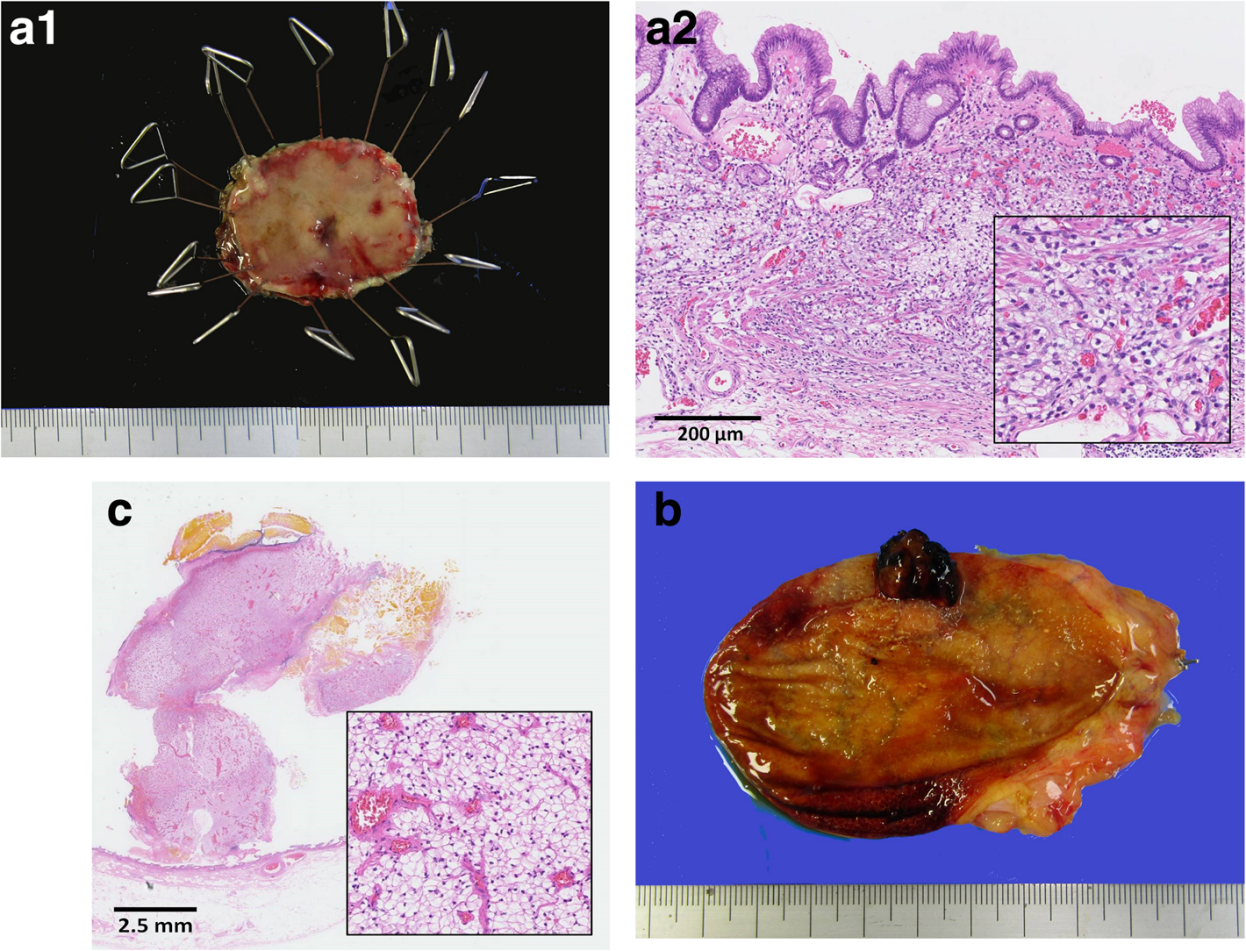
**a** Representative examples of the gastric tumor under low power view and the view of × 10 and × 20 objective lens using hematoxylin and eosin (H&E) stain. Tumor focally proliferates in the mucosal layer, with negative lymphovascular invasion. The surgical margin is all negative. The tumor contained glycogen-rich and acidophilic cytoplasm exhibiting honeycomb-formed or trabecular structure, resembling the patient’s known RCC. **b** Representative examples of the gallbladder tumor under low power view. **c** The tumor cell is similar to the gastric one and also proliferates in the mucosal layer

In February 2018, a gastric wedge resection via laparoscopic and endoscopic cooperative surgery (LECS) technique was applied to the gastric tumor, and laparoscopic cholecystectomy to the gallbladder tumor was simultaneously performed (Fig. 3). The operation lasted 190 min with little intraoperative blood loss. Intraoperative pathologic diagnosis was not performed in this case. The hospitalization period after surgery was not eventful, and the patient was discharged on postoperative day 7. Histological examination confirmed that the tumors of the stomach and gallbladder were both metastatic RCC. Immunohistochemical staining was strongly positive for CAM 5.2 and vimentin, supporting the diagnosis. Macro- and microscopic findings are shown in Fig. 4. Thereafter, the patient required examination every 3 months without the use of anticancer agents and has survived without relapse to 12 months after the surgery.

## Conclusions

According to analysis obtained from autopsy specimens [2], the occurrence of metastasis to the stomach constitutes 5.4% of the total number of metastases from solid malignancies. Particularly, melanoma, lung cancer, breast cancer, and esophageal cancer are well known as common primary tumors; however, RCC is not. Likewise, in other large series studies [3, 4], RCC metastasis to the stomach is rarely mentioned; therefore, the frequency of occurrence is not yet clarified.

Although typical sites of RCC metastasis are the lung, bones, liver, lymph nodes, or adrenal glands, RCC metastasis to the stomach is quite a rare condition, and few case reports have been presented so far. When a PubMed search was performed to identify case reports written in English and published within the past three decades using the keywords “renal cell carcinoma,” “metastasis,” and “stomach,” only 31 case reports (excluding our case) and case series reports were detected [5–35]. Of those, as a larger case series repot, Sakurai et al. [26] entered details of 22 cases reported during a period from 2008 to 2012. Rita et al. [28] described the clinicopathological features and focused endoscopic therapy of 44 cases during a period from 1980 to 2014.

On the other hand, when searching the literature written in Japanese, 24 cases regarding gastric metastasis from RCC were reported in the past three decades, and Oizumi et al. [36] reviewed these clinicopathological features. In summary, most patients were male (92%) and the mean age was 65.9 (range 40–77, median 69). The time interval to detection of gastric metastasis from initial nephrectomy was 73 months (range 0–228, average 75, median 59). Most patients (71%) had remote metastasis of other organs, and only 7 patients (29%) were diagnosed as solitary gastric metastasis.

Our case exhibited simultaneous metastasis to the gallbladder as well. RCC metastasis to the gallbladder has been frequently reported comparatively; however, these findings are still a rare site of metastasis. To the best of our knowledge, our case is the first report to address simultaneous multifocal metastasis of the combination of stomach and gallbladder. As a similar situation to our present case, Kongnyuy et al. [32] recently reported simultaneous and multifocal metastases to ipsilateral left testes, bladder, and stomach from RCC, 7 years after a left nephrectomy.

Recent developments in laparoscopic surgical techniques and instruments enable a minimally invasive metastasectomy. Some researchers report laparoscopic total pancreatectomy for multiple RCC metastases in the pancreas was beneficial to patients [37]. In our present case, gastric tumors were resected by a LECS technique. LECS is now the preferred treatment for gastric submucosal tumors [38] or duodenal tumors [39]; however, LECS experience in gastric metastatic tumors is limited due to its rarity. In our present case, LECS was adopted for the gastric tumor, as EUS suggested it potentially invaded to the submucosal layer. In a review describing endoscopic treatment for RCC gastric metastasis [28], surgical resection was performed in 39.5% of the patients. On the other hand, Japanese research describes treatment approaches for 22 patients with RCC gastric metastasis [36] and reports that endoscopic, surgical, and best supportive care were selected for 12, 8, and 4 patients, respectively. Although surgical treatment for these patients seemed to be highly limited, owing to the coexistence of other organ metastasis, interestingly, the patients who received a complete metastasectomy achieved a longer survival period. In the treatment for local metastases from RCC, no general guideline has yet reached consensus. In a recent systematic review, Dabestani et al. [40] concluded that complete metastasectomy substantially benefits patients’ survival and symptom palliation, with the assumption that their results were led by the retrospective studies included not those well-designed. Further analysis is needed to support our approach.

In summary, we experienced a rare case of simultaneous multifocal metastases of the stomach and gallbladder from RCC. Although it is still being discussed, for patients with locally resectable RCC metastases, complete metastasectomy may bring long-term tumor control. Moreover, LECS for the gastric metastasis is a reasonable approach with minimal invasiveness and an oncologically feasible outcome.

## References

[1] Cancer Facts and Figures 2018. Atlanta, Georgia: American Cancer Society, 2018. Available online. Last accessed September 12, 2018.

[2] Oda I, Kondo H, Yamao T, Saito D, Ono H, Gotoda T (2001). Metastatic tumors to the stomach: analysis of 54 patients diagnosed at endoscopy and 347 autopsy cases. Endoscopy.

[3] Menuck LS, Amberg JR (1975). Metastatic disease involving the stomach. Am J Dig Dis.

[4] Green LK (1990). Hematogenous metastases to the stomach. A review of 67 cases. Cancer.

[5] Sullivan WG, Cabot EB, Donohue RE (1980). Metastatic renal cell carcinoma to stomach. Urology.

[6] Vergara V, Marucci M, Marcarino C, Brunello F, Capussotti L (1993). Metastatic involvement of the pancreas from renal cell carcinoma treated by surgery. Ital J Gastroenterol.

[7] Odori T, Tsuboi Y, Katoh K, Yamada K, Morita K, Ohara A (1998). A solitary hematogenous metastasis to the gastric wall from renal cell carcinoma four years after radical nephrectomy. J Clin Gastroenterol.

[8] Picchio M, Paioletti A, Santini E, Iacoponi S, Cordahi M (2000). Gastric metastasis from renal cell carcinoma fourteen years after radical nephrectomy. Acta Chir Belg.

[9] Mascarenhas B, Konety B, Rubin JT (2001). Recurrent metastatic renal cell carcinoma presenting as a bleeding gastric ulcer after a complete response to high-dose interleukin-2 treatment. Urology.

[10] Riviello C, Tanini I, Cipriani G, Pantaleo P, Nozzoli C, Poma A (2006). Unusual gastric and pancreatic metastatic renal cell carcinoma presentation 10 years after surgery and immunotherapy: a case report and a review of literature. World J Gastroenterol.

[11] Saidi RF, Remine SG (2007). Isolated gastric metastasis from renal cell carcinoma 10 years after radical nephrectomy. J Gastroenterol Hepatol.

[12] Pezzoli A, Matarese V, Boccia S, Simone L, Gullini S (2007). Gastrointestinal bleeding from gastric metastasis of renal cell carcinoma, treated by endoscopic polypectomy. Endoscopy.

[13] Pollheimer MJ, Hinterleitner TA, Pollheimer VS, Schlemmer A, Langner C (2008). Renal cell carcinoma metastatic to the stomach: single-centre experience and literature review. BJU Int.

[14] Kibria R, Sharma K, Ali SA, Rao P (2009). Upper gastrointestinal bleeding revealing the stomach metastases of renal cell carcinoma. J Gastrointest Cancer.

[15] Yamamoto D, Hamada Y, Okazaki S, Kawakami K, Kanzaki S, Yamamoto C (2009). Metastatic gastric tumor from renal cell carcinoma. Gastric Cancer.

[16] Sugasawa H, Ichikura T, Ono S, Tsujimoto H, Hiraki S, Sakamoto N (2010). Isolated gastric metastasis from renal cell carcinoma 19 years after radical nephrectomy. Int J Clin Oncol.

[17] Eslick GD, Kalantar JS (2011). Gastric metastasis in renal cell carcinoma: a case report and systematic review. J Gastrointest Cancer..

[18] Tiwari P, Tiwari A, Vijay M, Kumar S, Kundu AK (2010). Upper gastro-intestinal bleeding - rare presentation of renal cell carcinoma. Urol Ann.

[19] Senadhi V, Jani N, Erlich R (2010). Metastatic renal cell cancer and a gastric mass: an unusual finding. Case Rep Gastroenterol.

[20] Cruz A, Ramírez LM, Sánchez E, Ruiz M, Moreno I, López J (2011). Gastric metastasis from renal cancer six years after nephrectomy. Rev Esp Enferm Dig.

[21] Namikawa T, Iwabu J, Kitagawa H, Okabayashi T, Kobayashi M, Hanazaki K (2012). Solitary gastric metastasis from a renal cell carcinoma, presenting 23 years after radical nephrectomy. Endoscopy.

[22] Siriwardana HP, Harvey MH, Kadirkamanathan SS, Tang B, Kamel D, Radzioch R (2012). Endoscopic mucosal resection of a solitary metastatic tumor in the stomach: a case report. Surg Laparosc Endosc Percutan Tech.

[23] Gómez-de-la-Cuesta S, Fernández-Salazar L, Velayos-Jiménez B, Macho-Conesa A, Ruiz-Rebollo L, Aller-de-la-Fuente R (2012). Gastric metastasis from renal cell carcinoma. Rev Esp Enferm Dig.

[24] Kim MY, Jung HY, Choi KD, Song HJ, Lee JH, Kim DH (2012). Solitary synchronous metastatic gastric cancer arising from t1b renal cell carcinoma: a case report and systematic review. Gut Liver.

[25] Namikawa T, Hanazaki K (2014). Clinicopathological features and treatment outcomes of metastatic tumors in the stomach. Surg Today.

[26] Sakurai K, Muguruma K, Yamazoe S, Kimura K, Toyokawa T, Amano R (2014). Gastric metastasis from renal cell carcinoma with gastrointestinal bleeding: a case report and review of the literature. Int Surg.

[27] Onorati M, Petracco G, Uboldi P, Redaelli DG, Romagnoli S, Albertoni M (2013). A solitary polypoid gastric metastasis 20 years after renal cell carcinoma: an event to be considered, and a brief review of the literature. Pathologica.

[28] Rita H, Isabel A, Iolanda C, Alexander H, Pedro C, Liliana C (2014). Treatment of gastric metastases from renal cell carcinoma with endoscopic therapy. Clin J Gastroenterol.

[29] Kumcu E, Gönültas M, Ünverdǐ H, Hücümenoğlu S (2014). Gastric metastasis of a renal cell carcinoma presenting as a polypoid mass. Endoscopy.

[30] Costa TN, Takeda FR, Ribeiro U, Cecconello I (2014). Palliative laparoscopic resection of renal cell carcinoma metastatic to the stomach: report of a case. World J Surg Oncol.

[31] Forman J, Marshak J, Tseng YA, Friedel DM, Grendell J (2015). Image of the month: gastric metastasis of renal clear cell carcinoma. Am J Gastroenterol.

[32] Kongnyuy M, Lawindy S, Martinez D, Parker J, Hall M (2016). A rare case of the simultaneous, multifocal, metastatic renal cell carcinoma to the ipsilateral left testes, bladder, and stomach. Case Rep Urol.

[33] O'Reilly MK, Sugrue G, Han-Suyin K, Fenlon H. Radiological, pathological and gross correlation of an isolated renal cell carcinoma metastasis to the stomach. BMJ Case Rep. 2017;2017:bcr-2017-220469. 10.1136/bcr-2017-220469 PMC5623286 28478393

[34] Namikawa T, Munekage E, Ogawa M, Oki T, Munekage M, Maeda H (2017). Clinical presentation and treatment of gastric metastasis from other malignancies of solid organs. Biomed Rep.

[35] Hemmerich A, Shaar M, Burbridge R, Guy CD, McCall SJ, Cardona DM (2018). Metastatic renal cell carcinoma as solitary subcentimeter polypoid gastric mucosal lesions: clinicopathologic analysis of five cases. Gastroenterology Res.

[36] Oizumi Y, Mieno H, Moriya H, Hosoda K, Yamashita K, Watanabe M (2016). Gastric metastasis from renal cell carcinoma 29 months after radical nephrectomy. J Jpn Surg Assoc.

[37] Choi YJ, Lee JH, Lee CR, Han WK, Kang CM, Lee WJ (2017). Laparoscopic total pancreatectomy for multiple metastasis of renal cell carcinoma of the pancreas: a case report and literature review. Ann Hepatobiliary Pancreat Surg.

[38] Hiki N, Nunobe S, Matsuda T, Hirasawa T, Yamamoto Y, Yamaguchi T (2015). Laparoscopic endoscopic cooperative surgery. Dig Endosc.

[39] Ichikawa D, Komatsu S, Dohi O, Naito Y, Kosuga T, Kamada K (2016). Laparoscopic and endoscopic co-operative surgery for non-ampullary duodenal tumors. World J Gastroenterol.

[40] Dabestani S, Marconi L, Hofmann F, Stewart F, Lam TB, Canfield SE (2014). Local treatments for metastases of renal cell carcinoma: a systematic review. Lancet Oncol.

